# Resistance Breeding of Common Bean Shapes the Physiology of the Rhizosphere Microbiome

**DOI:** 10.3389/fmicb.2019.02252

**Published:** 2019-10-01

**Authors:** Lucas William Mendes, Miriam Gonçalves de Chaves, Mariley de Cassia Fonseca, Rodrigo Mendes, Jos M. Raaijmakers, Siu Mui Tsai

**Affiliations:** ^1^Cell and Molecular Biology Laboratory, Center for Nuclear Energy in Agriculture CENA, University of São Paulo, Piracicaba, Brazil; ^2^Department of Microbial Ecology, Netherlands Institute of Ecology (NIOO-KNAW), Wageningen, Netherlands; ^3^Embrapa Meio Ambiente, Jaguariúna, Brazil; ^4^Institute of Biology Leiden, Leiden University, Leiden, Netherlands

**Keywords:** plant-microbe interactions, metatranscriptome, Biolog EcoPlate, carbohydrate metabolism, nutrient metabolism, resistome

## Abstract

The taxonomically diverse rhizosphere microbiome contributes to plant nutrition, growth and health, including protection against soil-borne pathogens. We previously showed that breeding for *Fusarium*-resistance in common bean changed the rhizosphere microbiome composition and functioning. Here, we assessed the impact of *Fusarium*-resistance breeding in common bean on microbiome physiology. Combined with metatranscriptome data, community-level physiological profiling by Biolog EcoPlate analyses revealed that the rhizosphere microbiome of the *Fusarium*-resistant accession was distinctly different from that of the *Fusarium*-susceptible accession, with higher consumption of amino acids and amines, higher metabolism of xylanase and sialidase, and higher expression of genes associated with nitrogen, phosphorus and iron metabolism. The resistome analysis indicates higher expression of soxR, which is involved in protecting bacteria against oxidative stress induced by a pathogen invasion. These results further support our hypothesis that breeding for resistance has unintentionally shaped the assembly and activity of the rhizobacterial community toward a higher abundance of specific rhizosphere competent bacterial taxa that can provide complementary protection against fungal root infections.

## Introduction

The rhizosphere, i.e., the zone of soil surrounding and influenced by the plant root, is considered one of the most dynamic ecosystems, constituting a hotspot of microbial activity. The complex processes in the rhizosphere lead to local variations in chemical make-up and concomitantly affect the taxonomic composition of the rhizosphere microbiome (Moe, 2013). The rhizosphere microbiome, referred to as the totality of microorganisms, their genomes and interactions, plays a key role in plant functioning, influencing its physiology and development, facilitating nutrient acquisition in exchange for photosynthetically fixed carbon (Philippot et al., 2013). The exudation of organic compounds by plant roots is considered the major basis for plant-rhizosphere interactions. In general, plant roots release up to 20% of fixed carbon and 15% of nitrogen, including simple molecules such as sugars, organic acids, secondary metabolites, and complex polymers such as mucilage (Sasse et al., 2018). The pattern of exudation is defined by the genotype of the host and can differ even between cultivars of the same species and between plant developmental stages (Chaparro et al., 2014; Mönchgesang et al., 2016). Hence, small changes in the plant genome could lead to a different exudation pattern and affect the rhizosphere microbiome.

The rhizosphere microbiome also plays a fundamental role in plant protection against soil-borne pathogens (Mendes et al., 2011; Berendsen et al., 2012; Santhanam et al., 2015; Chapelle et al., 2016; Carrión et al., 2018). Microbes in the rhizosphere can induce systemic resistance in plants and/or suppress root colonization by soil-borne pathogens (Lugtenberg and Kamilova, 2009). In this context, Wei et al. (2015) investigated the ability of the plant pathogen *Ralstonia solanacearum* to invade rhizobacterial communities that differed based on carbon competition networks. They showed that rhizobacterial communities with a clear niche overlap with the pathogen were better able to suppress infection. This is in line with the general conceptual ecological framework that a more diverse community is more prone to fend-off invading pathogens (van Elsas et al., 2012; Mallon et al., 2015). For several crop species, breeding for disease-resistant cultivars is the most efficient way to control soil-borne diseases, as is the case for Fusarium wilt of common bean (*Phaseolus vulgaris* L.) caused by the fungal root pathogen *Fusarium oxysporum* f. sp. *phaseoli* (*Fox*). In previous studies (Mendes et al., 2018a), we compared the rhizosphere microbiome composition of common bean cultivars with different levels of resistance to *Fox* and showed that specific beneficial rhizobacterial genera and functional traits were more abundant in the rhizosphere of the *Fox*-resistant bean cultivar. Several of these functional traits may help reinforce protection of the bean roots against *Fox*-infections.

Here, we performed an integrated analysis of the metabolic capabilities of the rhizobacterial community of the *Fox*-resistant and susceptible bean cultivars grown in the Amazon Dark Earth (ADE) soil. The ADE soil encompasses anthropogenic horizons built-up by the Pre-Colombian Indians between 500 and 8,700 years ago, and is characterized by high fertility and high microbial diversity (Germano et al., 2012). Considering putative differences in the quantity and quality of root exudates, we hypothesize that the *Fox*-resistant bean cultivar selects for a physiologically different rhizobacterial community. To this end, we conducted community-level physiological profiling using Biolog EcoPlate assays and assessed microbial metabolism of carbohydrates and other nutrients using metatranscriptome sequencing. We also assessed the rhizosphere resistome of the two bean cultivars, i.e., the collective set of antibiotic resistance genes in the microbiome (Wright, 2007). This allowed us to investigate the effects of *Fox*-resistance breeding on the rhizosphere microbiome physiology and to identify potential microbial traits that may help the plant to fend-off pathogen infections.

## Materials and Methods

### Greenhouse Experiment

The two common bean cultivars IAC Milenio (*Fox*-resistant; Carbonell et al., 2014) and IAC Alvorada (*Fox*-susceptible; Carbonell et al., 2008), were grown in mesocosm experiment with ADE soil. The *Fox*-resistant IAC Milenio is derived from a cross between two other cultivars, one being a sibling line of the susceptible IAC Alvorada, reinforcing the small genomic differences between them (Carbonell et al., 2014). For the experiments, ceramic pots (30 cm high × 20 cm diameter) with a stone layer of 5 cm on the bottom were filled with approximately 8 kg of ADE soil. Each cultivar was grown in three independent pots with three bean seeds each. We kept three pots without plants, which were considered as bulk soil. The seeds germinated at 28/19°C (day/night) with a 12-h photoperiod. The moisture and temperature were regularly monitored to allow optimal growth conditions for the plants. Plants were collected at R1 development stage (early flowering) and the roots with attached soil were removed from the pots and transported on ice to the laboratory. The roots were shaken to remove loosely adhering soil. The firmly attached soil was collected with sterile brushes and considered to be the rhizosphere soil. These samples were used for Biolog EcoPlate assays. Considering the natural occurrence of Fusarium in soils, we conducted the experiments without the inoculation of the fungi in order to resemble field conditions and only health plants were sampled.

### Biolog EcoPlate Assays

To determine substrate utilization by the microbial community from the bulk soil and the rhizosphere of the two cultivars, we used Biolog EcoPlates^TM^ (Biolog, Inc., Hayward, CA, United States). For this, 1 g of fresh bulk or rhizosphere soil was suspended in 9 mL of 0.85% sterile NaCl (dilution to 10^–1^) and shaken at 25°C for 30 min at 150 rpm. After 10 min settling, the suspension was diluted 100- and 1000-fold. A volume of 125 μl of the 1000-fold dilution was pipetted into the microplate wells and the plates were incubated at 25°C in the dark for 7 days. One EcoPlate was used for each soil replicate [(1 bulk soil + 2 rhizosphere) × 5 replicates] totaling 15 plates. All measurements were done in three technical replicates per plate, as the 96-well system contains three times the 31 carbon sources and three times the control. For analysis, we used the average of the three replicates per plate. The increasing intensity of purple color was followed over time by measuring OD_590_ every 24 h for a total of 168 h (7 days) in a Cary 50 Microplate Reader (Varian, Inc., Walnut Creek, CA, United States). The OD values were subjected to data corrections prior to evaluation, including first the subtraction of the OD_590_ value of the control well (water only) followed by subtraction of the initial OD value of each well measured right after filling the wells with the soil suspension (considered here day 1) to eliminate the effect of soil particles on the read-out of the OD values. Negative values were set to zero. The data analysis was performed on the OD-values obtained after 168 h incubation (seventh day of measurements). The 168 h absorbance values were used for calculating the average well color development (AWCD – an average of all substrates) and the substrate average well color development (SAWCD – an average of the substrates divided in guilds, as described below), according to Feigl et al. (2017), as follow: AWCD = Σ OD_*i*_/N, where OD_*i*_ is the correct OD value of each substrate containing well and N is the number of substrates, in this case *N* = 31; SAWCD = Σ OD_*i*_/N, where, in this case, N is the number of substrates in each category. The substrate categories were defined into six groups representing different substrate guilds according to Sala et al. (2010), as follows: amino acids (L-arginine, L-asparagine, L-phenylalanine, L-serine, glycyl-L-glutamic acid, L-threonine), amines (phenylethylamine, putrescine), carbohydrates (D-mannitol, glucose-1-phosphate, D, L-alpha-glycerol phosphate, beta-methyl-D-glucoside, D-galactonic acid-gamma-lactone, i-erythritol, D-xylose, N-acetyl-D-glucosamine, D-cellobiose, alpha-D-lactose), carboxylic acids (D-glucosaminic acid, D-malic acid, itaconic acid, pyruvic acid methyl ester, D-galacturonic acid, alpha-ketobutiryc acid, gamma- hydroxybutyric acid), phenolic compound (2-hydroxy benzoic acid, 4-hydroxy benzoic acid) and polymers (Tween 40, Tween 80, alpha-cyclodextrin, glycogen). Principal component analysis (PCA) was used to compare the community substrate utilization using the software Canoco 4.5 (Biometrics, Wageningen, Netherlands). Shannon diversity index was calculated based on the absorbance values at 168 h. Statistical data analyses were performed using one-way analysis of variance and Tukey’s test. Diversity index and statistical analyses were calculated with Past 3 (Hammer et al., 2001).

### Metatranscriptome Data and Analysis

In this study, we further analyzed the metatranscriptome data generated in the experiment previously conducted in our group (Mendes et al., 2018a) and available in MG-RAST in the project ‘Common Bean Rhizosphere Metatranscriptome’ (mgp20659) focusing on the two most contrasting cultivars, i.e., IAC Milenio and IAC Alvorada. The experimental design and sampling was identical to the one used here to collect data for the Biolog EcoPlates. We focused the analysis on carbohydrate and nutrient metabolism, and on the resistome. The screening of the datasets was performed with MEGAN6 (Huson et al., 2016) by providing the alignments resulting from DIAMOND (Buchfink et al., 2015) against the NCBI-NR database. The reads count was normalized to the smallest number of reads (Huson et al., 2016). Functional profiling related to metabolism of carbohydrate, nitrogen, phosphorus, potassium, sulfur and iron was determined with the SEED database (Overbeek et al., 2005), and the generated matrix was exported for further statistical analysis. For annotation of carbohydrate-active enzymes (CAZy) and antibiotic resistance genes (ARGs), we first performed the gene calling using PRODIGAL (Hyatt et al., 2010) to identify open read frames (ORFs) in the reads. The annotation was performed with HMMSCAN (Eddy, 2011) using the Hidden Markov Model (HMM) profiles. For carbohydrate-active enzyme annotation we used the HMM profile available in the dbCAN database (Yin et al., 2012). For the resistome annotation, we used the HMM profile available in RESFAM database, a curated bank of protein families confirmed for antibiotic resistance function and organized by ontology (Gibson et al., 2015).

In order to compare the structure of the functional profiles, we conducted principal component analysis (PCA) with the software Canoco 4.5. We used permutational multivariate analysis of variance (PERMANOVA) (Anderson, 2001) to confirm the differences of the structure of the functional profiles among treatments. Alpha diversity index was calculated from a matrix of richness of functions using the Shannon’s index. PERMANOVA and alpha diversity index were calculated with the software PAST 3 (Hammer et al., 2001). In order to visualize the statistically different microbial functional profiles among the treatments, we used the Statistical Analysis of Metagenome Profile software (STAMP) (Parks et al., 2014). For this, matrices of specific metabolisms were generated and the level of gene expression was compared based on *P*-values calculated using the two-sided Welch’s *t*-test and correction was made using Benjamini-Hochberg false discovery rate (Benjamini and Hochberg, 1995).

## Results and Discussion

### Community-Level Physiological Profiles (CLPP) of the Rhizosphere Microbiome

**FIGURE 1:**
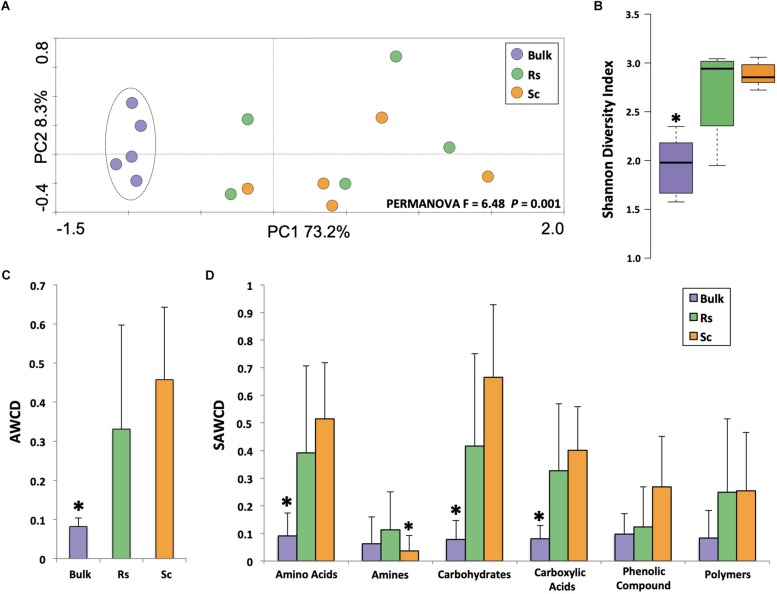
Analysis of the community-level physiological profiling (CLPP) based on Biolog EcoPlates measurements for bulk soil and rhizosphere. **(A)** Principal component analysis of the CLPP. The dashed line indicates significant cluster based on PERMANOVA (*P* < 0.05). **(B)** Shannon diversity index based on CLPP profile at 168 h of incubation. **(C)** Average well color development (AWCD) at 168 h of incubation. **(D)** Substrate average well color development (SAWDC) at 168 h of incubation. Asterisks indicate significant differences based on Tukey’s test (*P* < 0.05). Bulk, bulk soil; Rs, Resistant cultivar; Sc, Susceptible cultivar.

CLPP analysis revealed a clear distinction between the carbon degradation profiles of the microbial communities of the bulk soil and the rhizosphere (Figure 1A, PERMANOVA *F* = 6.48, *P* = 0.001), with a higher metabolic activity in the rhizosphere than in the bulk soil based on the Shannon diversity index (Figure 1B). The AWCD (average well color development – an average of all substrates) analysis also showed that the rhizosphere community utilized the carbon sources more efficiently overall (Figure 1C). Regarding the categorized substrate sources (SAWCD analysis), our results showed that amino acids, carbohydrates and carboxylic acids were utilized more efficiently by the rhizosphere microbes than by the bulk soil microbes (Figure 1D). From the 31 carbon sources, both rhizosphere communities used 12 substrates significantly more than the microbes from the bulk soil, namely D-cellobiose, beta-methyl-D-glucoside, i-erythritol, D-mannitol, glucose-1-phosphate, D-galactonic acid gama-lactone, tween 80, L-arginine, L-phenylalanine, L-serine, pyruvic acid methyl ester, and D-malic acid (*P* < 0.05, Supplementary Figure 1). These results suggest that the microbial community of the rhizospheres of the two common bean accessions is metabolically more versatile than the microbial community from the bulk soil (Figure 1D).

Roots release low-molecular-mass compounds, such as amino acids, sugars and organic acids, that can represent up to 20% of the carbon allocated to the roots (Philippot et al., 2013). Although our CLPP analysis revealed a clear separation between rhizosphere and bulk soil, there was no clear overall distinction between the rhizospheres of the two contrasting bean cultivars. Although these cultivars are genetically related, even small changes in the genome may affect plant physiology and rhizosphere community assembly. In our previous studies (Mendes et al., 2018b) we showed that these two contrasting cultivars harbor distinct microbial communities. Analyzing the individual carbon sources, we found that the rhizosphere microbiome of the *Fox*-resistant accession consumed more D, L-alpha-glycerol phosphate, glycogen, L-threonine, phenylethyl-amine, putrescine, and D-galacturonic acids compared to the rhizosphere microbiome of the susceptible bean accession (*P* < 0.05, Supplementary Figure 1). D-galacturonic acid is a key component of pectin, a major constituent of plant cell walls that is released in the rhizosphere (Zhang et al., 2011). The high consumption of D-galacturonic acid in the rhizosphere of the *Fox*-resistant cultivar may be related to the deposition of extra cell wall layers, a mechanism of the resistant cultivar to restrict pathogen invasion of the root tissue (Pereira et al., 2013). Based on the individual analysis of 31 carbon sources, the microbiome of the resistant cultivar also showed a preference for amino acids and amines. The ability of the microbiome to use amino acids and amines as biological source of carbon and nitrogen may confer a selective advantage to the community to colonize the rhizosphere (Moe, 2013), which may explain the higher abundance of bacteria (based on 16S rRNA quantification) in the rhizosphere of the resistant compared to the susceptible cultivar (Mendes et al., 2018b). Interestingly, the dynamics of exudation and consumption of amino acids in the rhizosphere can alter key phenotypes related to microbial colonization and pathogenesis (Moe, 2013). For example, our previous data showed higher activity of phenazine production in the rhizosphere of the *Fox*-resistant cultivar (Mendes et al., 2018a), and although the effect of individual amino acids is variable, they generally stimulate phenazine production in *Pseudomonas* (van Rij et al., 2004; Sakhtah et al., 2013). In conclusion, the CLPP analysis revealed that the rhizosphere of the *Fox*-resistant cultivar exhibited distinct differences in the utilization of specific carbon sources in comparison with the bulk soil and the *Fox-*susceptible cultivar. These physiological differences, such as the greater utilization of amino acids, may in turn lead to increased densities of beneficial bacterial groups that express specific antifungal metabolites, such as phenazines, reaching the threshold to restrict pathogen growth and infections.

### Metabolism of Carbohydrates

**FIGURE 2:**
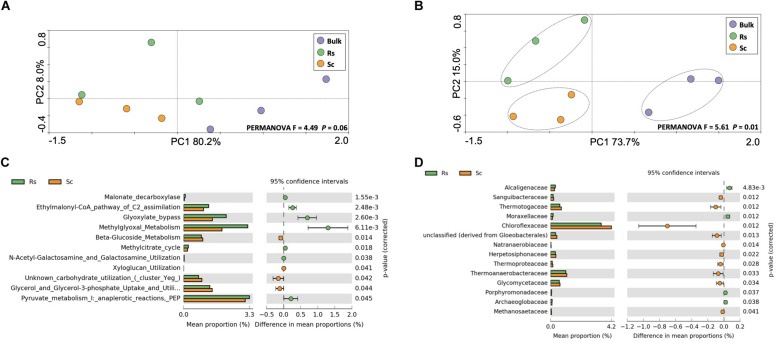
Analysis of the active community related to the metabolism of carbohydrate, performed on metatranscriptome sequences affiliated to the SEED database. Principal component analysis performed at **(A)** functional and **(B)** taxonomic level. The dashed lines indicate significant clusters based on PERMANOVA (*P* < 0.05). Differential expression of sequences affiliated to **(C)** the metabolism of carbohydrate at level 3 and **(D)** the taxonomic group related to the same metabolism. The differences between the treatments are based on Welch’s *t*-test with Benjamini-Hochberg correction (*P* < 0.05). Bulk, bulk soil; Rs, resistant cultivar; Sc, susceptible cultivar.

In order to complement the CCLP analysis, we analyzed the metabolism of carbohydrates using a molecular approach based on the metatranscriptome sequencing. Microorganisms require relatively small amounts of nitrogen for their structural processes, but do need large amounts of carbohydrates for their energy needs. When bacteria have access to easily digestible carbohydrates, they use sugars in preference over proteins (Bayne-Jones, 1936). Here, we hypothesized that the rhizosphere microbiome of the *Fox*-resistant bean accession exhibits a profile distinct from the *Fox*-susceptible accession. To address this hypothesis, we analyzed the metatranscriptome data based on taxonomy and functions related to carbohydrates metabolism. The results did not show a clear functional clustering of the samples based on treatment (Figure 2A). However, when the sequences of carbohydrate metabolism were taxonomically affiliated, the samples clustered according to the bean cultivar and bulk soil (Figure 2B, PERMANOVA *F* = 5.61, *P* = 0.01), suggesting a differentiation at the taxonomic level rather than the functional level. A higher taxonomic and functional diversity was found in the rhizosphere compared to the bulk soil, however, with no differences between the cultivars (Supplementary Figure 2). From the 100 functions identified at subsystem level 3 of the carbohydrate metabolism, 11 were differentially expressed in the rhizosphere microbiome (Figure 2C). The rhizospheric microbial community of the *Fox*-resistant cultivar showed more activity related to the central carbohydrate metabolism, organic acids and amino sugars, while the susceptible cultivar presented higher metabolism of di- and oligosaccharides, polysaccharides and sugar alcohol. Comparing the differential abundance of the active microbial groups at family level, the results revealed a different profile between the two bean cultivars, with the resistant presenting higher activity of families affiliated to Burkholderiales (Alcaligenaceae family) and Pseudomonadales (Moraxellaceae family) (Figure 2D). Several studies have depicted the Proteobacteria, in particular these two bacterial orders, as dominant members of the rhizosphere microbiome. This can be explained by their life style as r-strategists with the ability to utilize a broad range of root-derived carbon substrates (Philippot et al., 2013). This in turn confers a competitive advantage for these microbes in the rhizosphere keeping root-invasive pathogens in check (Wei et al., 2015).

**FIGURE 3:**
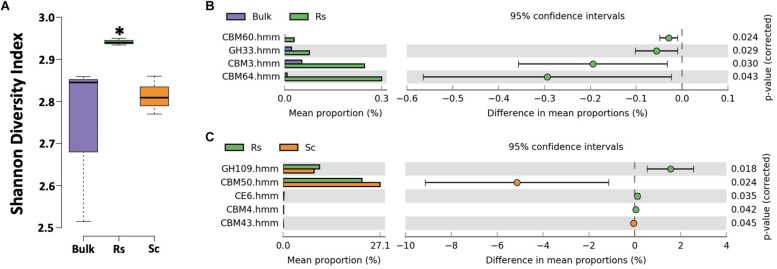
Analysis of the carbohydrate-active enzyme (CAZy) annotation using the dbCAN database. **(A)** Shannon diversity index based on the CAZy expression. Asterisk indicates significant difference based on Tukey’s test (*P* < 0.05). Differential expression of metatranscriptome sequences affiliated to CAZy **(B)** between bulk soil and *Fox*-resistant rhizosphere microbiome and **(C)** between the resistant and susceptible cultivar. The differences between the treatments are based on Welch’s *t*-test with Benjamini-Hochberg correction (*P* < 0.05). Bulk, bulk soil; Rs, resistant cultivar; Sc, susceptible cultivar.

To further explore carbohydrate metabolism by the rhizosphere microbiome, we conducted a carbohydrate-active enzyme (CAZy) annotation in dbCAN (Yin et al., 2012). The microbiome of the *Fox*-resistant cultivar exhibited a higher diversity of CAZy compared to the bulk soil (*P* < 0.05, Figure 3A). Interestingly, the CAZy diversity was also significant higher in the *Fox*-resistant cultivar compared to the *Fox*-susceptible (*P* < 0.05). More specifically, we observed higher expression of enzymes affiliated to xylanase (CBM60, CBM64, CBM4, CE6) and sialidase (GH33) in the rhizosphere of the *Fox*-resistant cultivar (Figures 3B,C). Xylanases degrade xylan, a major hemicellulose component in plant cell walls (Kosugi et al., 2002). This enzyme plays a key role in microbial degradation of plant matter into usable nutrients. As previously mentioned, the *Fox*-resistant plant presents an additional deposition of cell wall layers as a strategy to limit pathogen invasion, and this structural characteristic may explain the higher activity of this enzyme. Sialidases act in the catabolism of sialic acid-containing oligosaccharides. In microorganisms, sialidases can have a nutritional function as carbon and energy sources (Li and McClane, 2014). In a study with *Pseudomonas aeruginosa*, Pastoriza Gallego and Hulen (2006) showed that sialic acid contributed to bacterial adhesion, which may contribute to the higher abundance of this genus in the rhizosphere of *Fox*-resistant cultivar (Mendes et al., 2018b). In general, our results showed that the rhizosphere of the *Fox*-resistant cultivar presented higher diversity and expression of several enzymes, and this characteristic can be interpreted as a greater functional diversity of the microbial community in this niche (Kandeler et al., 2002).

### Metabolism of Nutrients

**FIGURE 4:**
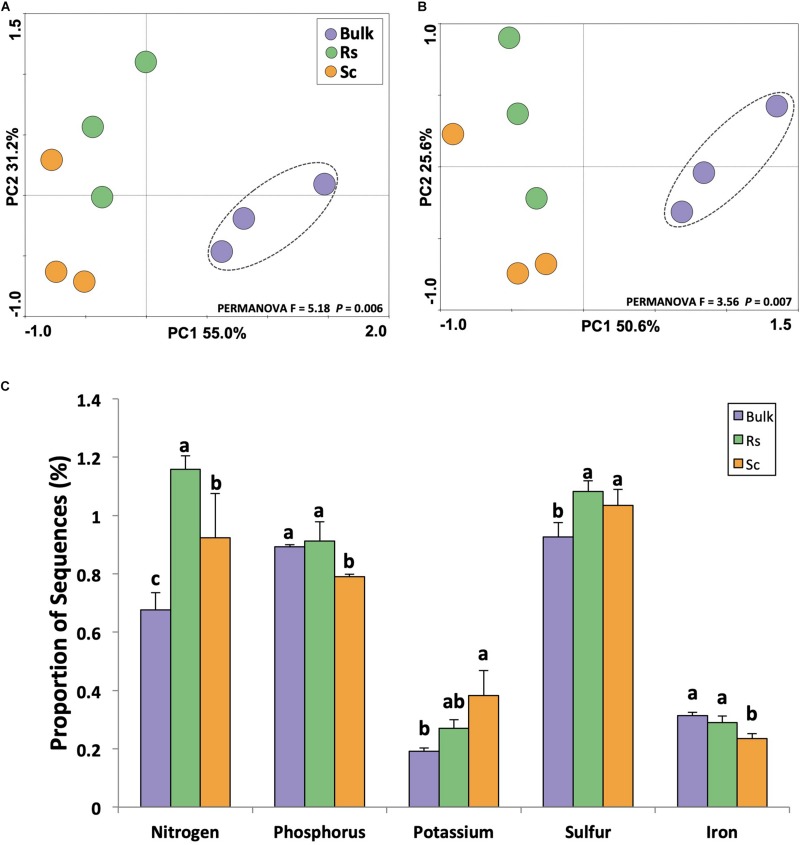
Analysis of the metatranscriptome sequences affiliated to the metabolism of nutrients using the SEED database. Principal component analysis performed at **(A)** functional and **(B)** taxonomic level. The dashed lines indicate significant clusters based on PERMANOVA (*P* < 0.05). **(C)** Differential expression of genes affiliated to the metabolism of nutrients based on the proportion of the sequences. Different lower case letters indicate significant differences between the treatments based on Tukey’s test (*P* < 0.05). Bulk, bulk soil; Rs, resistant cultivar; Sc, susceptible cultivar.

The rhizosphere microbiome plays an important role in nutrient cycling because it decomposes plant material and soil organic matter, releases inorganic nutrients, and affects nutrient availability by solubilization, chelation, oxidation and reduction (Marschner, 2007). Thus, the activity of the rhizosphere microbiome has a profound impact on plant growth and health, and healthier plants are more prone to fend off pathogen invasions (Spann and Schumann, 2009). In this context, we analyzed the metabolism of nitrogen, phosphorus, potassium, sulfur and iron based on the metatranscriptome sequences classified in the SEED database. The results indicated that both functional (Figure 4A) and taxonomic (Figure 4B) profiles associated with metabolism of these five selected nutrients were distinct between the rhizosphere and the bulk soil. Further analyzing the metabolism of these nutrients based on the abundance of sequences, we observed higher expression of genes related to the metabolism of nitrogen, phosphorus and iron in the rhizosphere of the *Fox*-resistant cultivar (Figure 4C). At a lower functional level, we observed that the rhizosphere microbiome of the *Fox*-resistant cultivar presented higher ammonia assimilation, metabolism of phosphate and potassium uptake system compared to the susceptible plant (Supplementary Figure 3). Mineral nutrition can exert a profound effect on fungal disease development by direct effect on the pathogen, or on plant growth and development, and on plant resistance mechanisms (Walters and Bingham, 2007). The first contact of the pathogen with the host plant, it is assumed to be nutrient starved, meaning that rapid assimilation of host nutrients is essential for successful infection (Gupta et al., 2013). In general, healthy plants are less susceptible to disease than nutrient-deficient plants, and the pathogen may reduce the nutrient availability to the plant increasing its susceptibility (Spann and Schumann, 2009). In this sense, higher metabolism of nutrients in the rhizosphere of the *Fox*-resistant cultivar could lead to high nutrient availability to the plant.

### Bulk Soil and Rhizosphere Resistome

**FIGURE 5:**
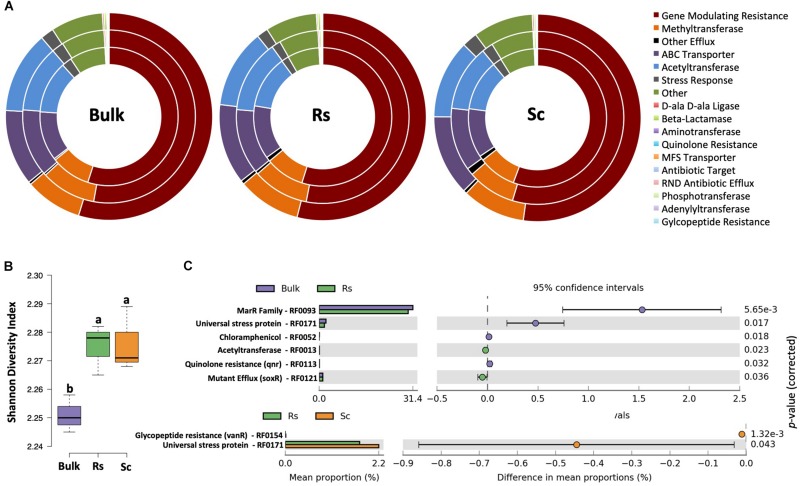
Analysis of the microbial resistome based on the affiliation of metatranscriptome sequences to the antibiotic resistance genes (ARGs) database. **(A)** Doughnut charts showing the classification of the sequences based on the resistome mechanism. **(B)** Shannon diversity index based on the expression of ARGs. Different lower case letters indicate significant differences between the treatments based on Tukey’s test (*P* < 0.05). **(C)** Differential expression of metatranscriptome sequences affiliated to ARGs comparing the *Fox*-resistant cultivar to the bulk soil and susceptible cultivar. The differences between the treatments are based on Welch’s *t*-test with Benjamini-Hochberg correction (*P* < 0.05). Bulk, bulk soil; Rs, resistant cultivar; Sc, susceptible cultivar.

In our previous study (Mendes et al., 2018b), we demonstrated that the rhizosphere microbiome of the *Fox-*resistant cultivar presented a higher microbial abundance and diversity than that of the *Fox-*susceptible bean. Given that antibiotic resistance genes (ARGs) play an important role in microbial competitiveness via detoxification of antimicrobial compounds (Nesme and Simonet, 2015), we screened ARGs using HMM models profiles that have been confirmed for antibiotic resistance function (Gibson et al., 2015). Our analysis revealed that the pattern of ARGs expressed in both bulk soil and rhizosphere was similar, with high expression of genes associated with mechanisms of ‘gene modulating resistance’, followed by ‘methyltransferase’, ‘acetyltransferase’ and ‘ABC transporters’ (Figure 5A). The methylation of specific rRNA nucleotides by the enzyme methyltransferase can prevent the binding of protein synthesis inhibitors to their target sites on the ribosome and lead to antibiotic resistance (Vester and Long, 2013). The chloramphenicol acetyltransferase enzyme (CAT) detoxifies the antibiotic chloramphenicol and is responsible for resistance in bacteria (Engel and Prockop, 1991). ABC transporters (ATP-biding cassette) are related to multidrug resistance by the transport of transmembrane xenobiotic molecules, including drugs, sugars, ions, amino acids and proteins (Greene et al., 2018). Interestingly, in our previous DNA-based work (Mendes et al., 2018b) we have shown a higher abundance of sequences affiliated to ABC transporters and protein secretion system in the rhizobacterial community of the *Fox*-resistant cultivar. Our data here revealed a higher diversity of ARGs in the bean rhizosphere than in the bulk soil (Figure 5B). Antibiotic resistance is advantageous to bacteria thriving in the rhizosphere, since the competition for space and resources is intense in this environment. The rhizosphere is indeed a battlefield where the complex root microbiome interacts with pathogens and influences the outcome of a pathogen infection (Raaijmakers et al., 2009). Although we found that most of the detected ARGs presented similar pattern of expression between the two bean cultivars, the susceptible plant exhibited higher expression of the genes related to glycopeptide resistance (vanR, RF0154) and universal stress protein (RF0171) (Figure 5C). Comparing the resistant cultivar with the bulk soil, we found higher expression of the genes related to acetyltransferase (RF0013) and mutant efflux (soxR, RF0121) (Figure 5C). The soxR is known to regulate genes involved in protecting bacteria against oxidative stress (Ha and Jin, 1999). The invasion of a pathogen, such as *F. oxysporum*, destabilize the homeostasis of the plant inducing the oxidative stress (Morkunas and Bednarski, 2008; Mohapatra and Mittra, 2017). In this sense, the higher expression of soxR in the rhizosphere of the *Fox*-resistant cultivar could help the plant to diminish the pathogen infection. In summary, our data revealed a higher diversity of ARGs in rhizosphere compared with the bulk soil, with specific differences between the two rhizospheres.

## Concluding Remarks

In our previous work (Mendes et al., 2018a), we have assessed the microbiome of four common bean cultivars with different levels of resistance to the soil-borne pathogen *Fusarium oxysporum* and we demonstrated that breeding for resistance unintentionally co-selected for changes in rhizosphere microbiome composition and functions that may act in concert to restrict root infections. In the present work, we further analyzed the effect of resistance breeding on the two most contrasting cultivars, i.e., the *Fox*-resistant IAC Milenio and the susceptible IAC Alvorada, focusing on the rhizosphere microbiome physiology through community-level physiological profiling and metatranscriptomics. Some mechanisms by which beneficial microorganisms protect crop plants from diseases include enhancement of overall vigor via nutrient mobilization and direct antagonism via antibiosis and competition (Wille et al., 2018). We found a preference for amino acid and amine utilization in the *Fox*-resistant rhizosphere microbiome. Regarding carbohydrate metabolism, we pinpointed rhizobacterial taxa associated with a higher expression of xylanase and sialidase enzymes in the *Fox*-resistant rhizosphere. Also, considering nutrient metabolism, the resistant cultivar exhibit higher expression of genes related to metabolism of nitrogen, phosphorus and iron. Considering that our study is limited by the use of two common bean cultivars and one soil type, to what extent different genotypes, soil types, plant development stages and inoculation of the pathogen could impact the rhizosphere microbiome physiology are subject of future experiments. Also, considering that the root exudate chemistry and microbial substrate preference are the main drivers of rhizosphere microbial community assembly (Zhalnina et al., 2018), future studies are needed to understand the effect of resistance breeding on quantitative and qualitative differences in root exudation and how these affect microbial community composition and activity. Disentangling the link between root exudation and microbial community assembly in the rhizosphere is essential to engineer and integrate root microbiomes in plant breeding programs focused on improved growth and tolerance to (a)biotic stresses.

## Data Availability Statement

The metatranscriptome data used in this study are available in MG-RAST server under the project ‘Common Bean Rhizosphere Metatranscriptome’ (mgp20659).

## Author Contributions

LM, ST, and RM designed the study. LM, MC, and MF conducted the experiments and obtained the data. LM analyzed the data. LM, JR, RM, and ST wrote the manuscript.

## Conflict of Interest

The authors declare that the research was conducted in the absence of any commercial or financial relationships that could be construed as a potential conflict of interest.
