# Supplementary material for: Resistance Breeding of Common Bean Shapes the Physiology of the Rhizosphere Microbiome
Source: Front Microbiol. 2019 Oct 1;10:2252. doi: 10.3389/fmicb.2019.02252 (PMC6779718; doi:10.3389/fmicb.2019.02252)
Supplement: Supplementary file 1 [file Data_Sheet_1.PDF]

SUPPLEMENTARY MATERIAL

Resistance breeding of common bean shapes the physiology of the rhizosphere microbiome

Lucas William Mendes, Miriam Gonçalves de Chaves, Mariley de Cassia da Fonseca, Rodrigo Mendes, Jos M Raaijmakers, Siu Mui Tsai

[1]Cell and Molecular Biology Laboratory, Center for Nuclear Energy in Agriculture CENA, University of Sao Paulo USP, 13416-000, Piracicaba, SP, Brazil; [2]Embrapa Meio Ambiente, 18020-000, Jaguariuna, Brazil. [3]Departament of Microbial Ecology, Netherlands Institute of Ecology NIOO-KNAW, 6708 PB, Wageningen, The Netherlands; [4]Institute of Biology, Leiden University, Leiden, the Netherlands.

Supplementary Figures 1 to 3

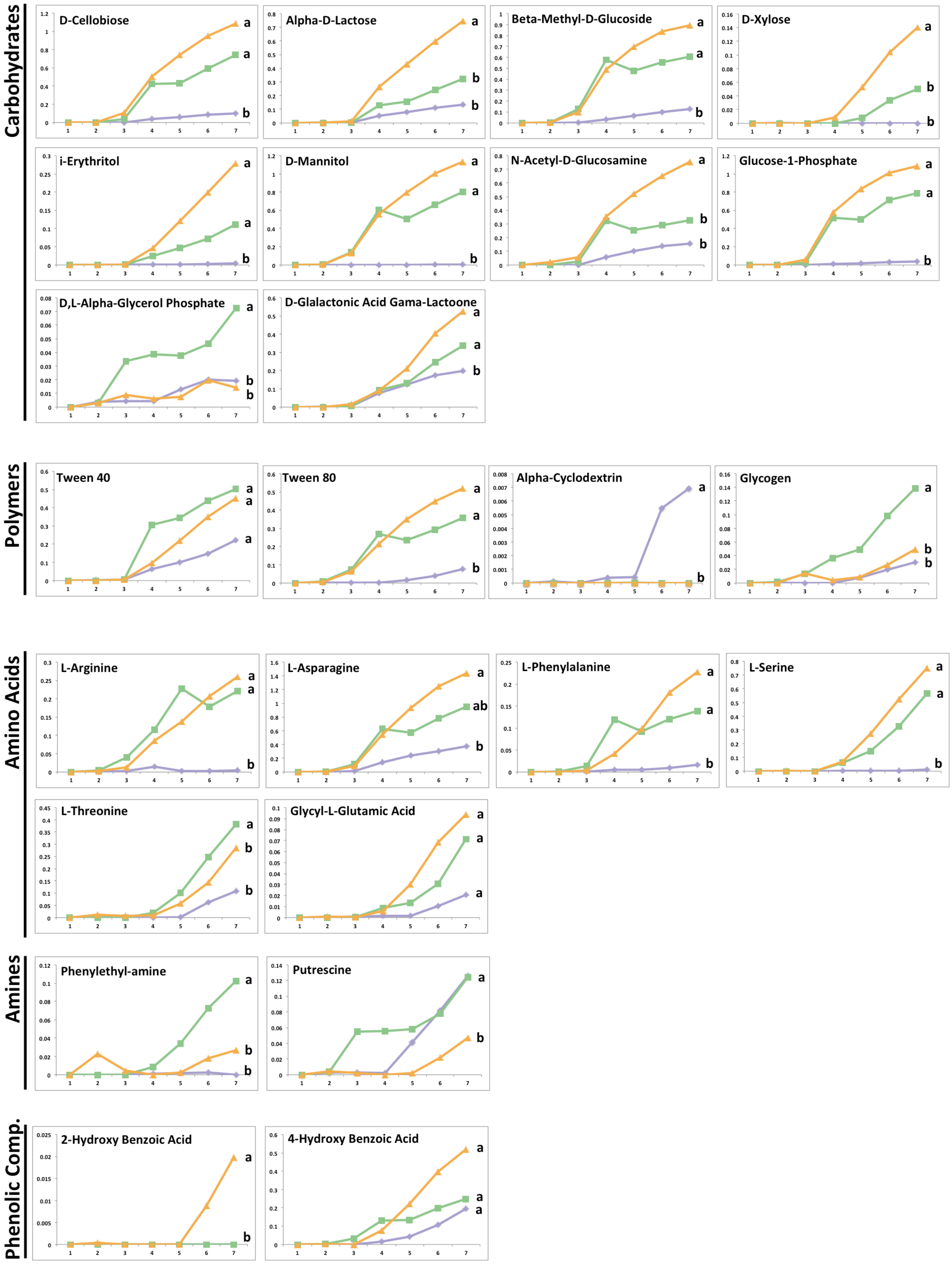
Carbohydrates
D-Cellobiose
Alpha-D-Lactose
Beta-Methyl-D-Glucoside
D-Xylose
i-Erythritol
D-Mannitol
N-Acetyl-D-Glucosamine
Glucose-1-Phosphate
D,L-Alpha-Glycerol Phosphate
D-Glalactonic Acid Gama-Lactoone
Polymers
Tween 40
Tween 80
Alpha-Cyclodextrin
Glycogen
Amino Acids
L-Arginine
L-Asparagine
L-Phenylalanine
L-Serine
L-Threonine
Glycyl-L-Glutamic Acid
Amines
Phenylethyl-amine
Putrescine
Phenolic Comp.
2-Hydroxy Benzoic Acid
4-Hydroxy Benzoic Acid

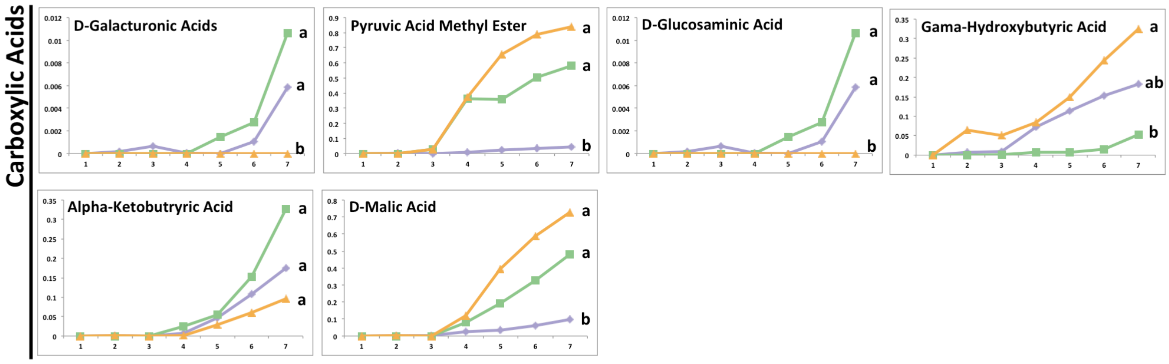

Supplementary Figure 1. Analysis of the community-level physiological profiling (CLPP) based on Biolog EcoPlates measurements. The X axis of the graph indicates the measurements at every 24h (1 day), while the Y axis indicates the average well color development values. Different lower case letters indicate significant differences between the treatments based on Tukey's test ($P < 0.05$) of the measurements at 168 h. Purple lines refer to bulk soil, green lines to fox-resistant cultivar, and yellow lines refer to susceptible cultivar.

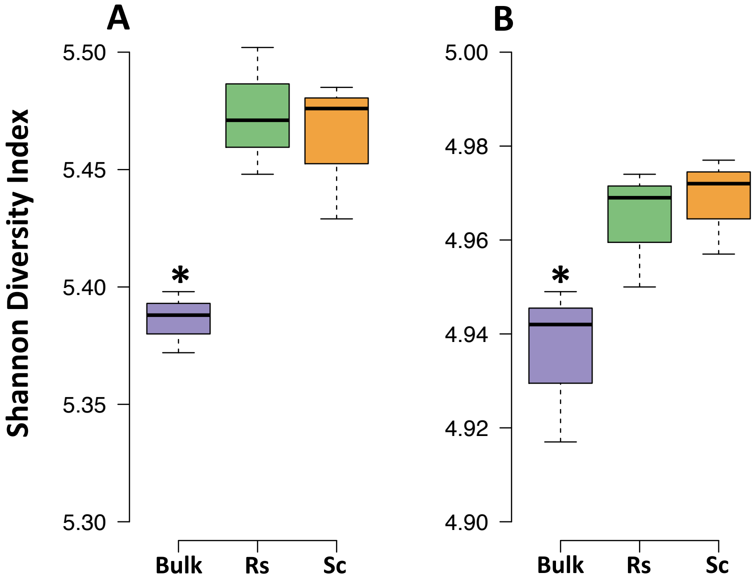

Supplementary Figure 2. Shannon diversity index based on the (A) functional and (B) taxonomic affiliation of the metatranscriptome sequences to the metabolism of carbohydrates using the SEED database. Asterisk indicates significant difference based on Tukey's test ($P < 0.05$). Bulk = bulk soil; Rs = Resistant cultivar; Sc = Susceptible cultivar.

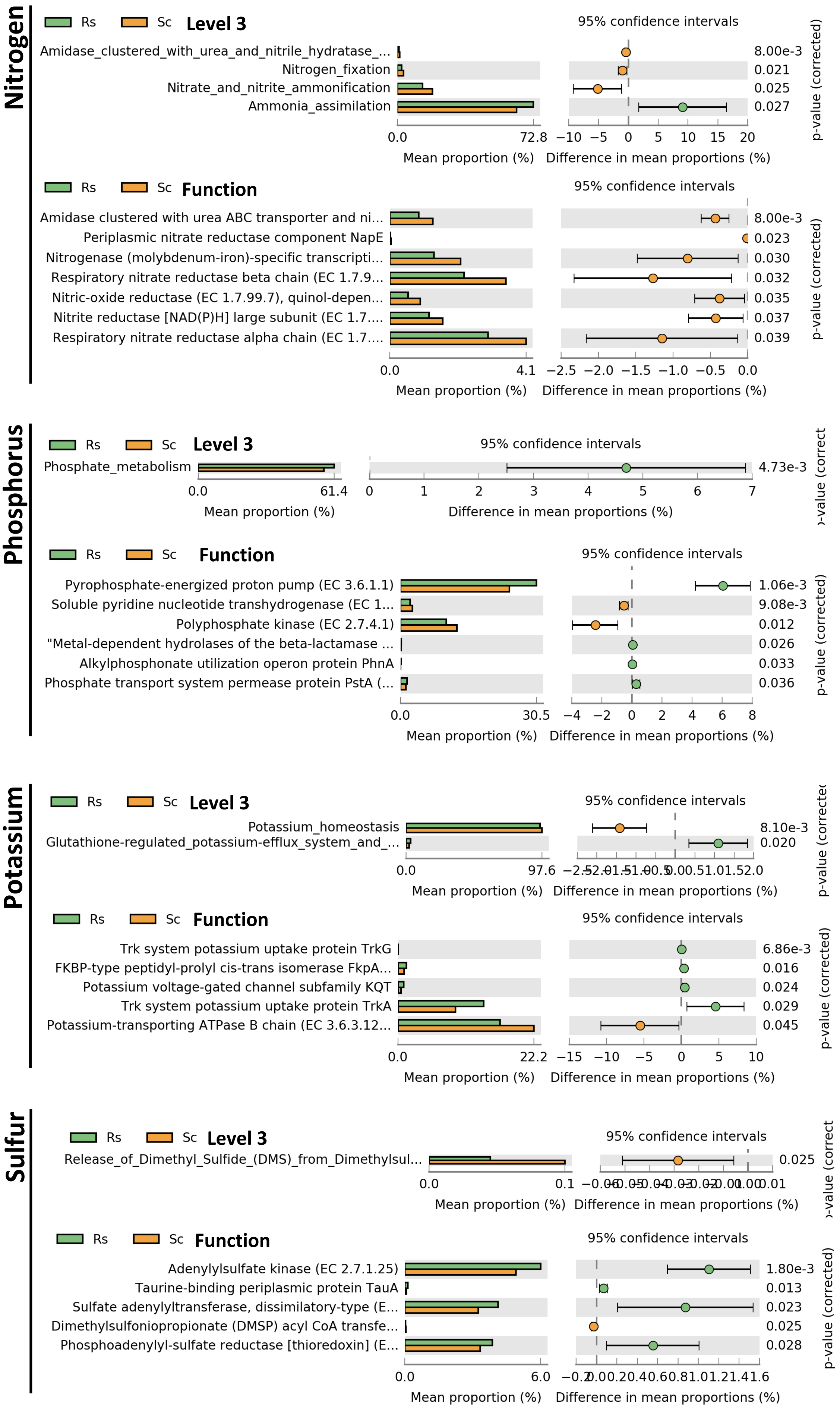
Nitrogen
Rs
Sc
Level 3
95% confidence intervals
Amidase_clustered_with_urea_and_nitrile_hydratase_...
Nitrogen_fixation
Nitrate_and_nitrite_ammonification
Ammonia_assimilation
8.00e-3
0.021
0.025
0.027
0.0
72.8
−10 −5 0 5 10 15 20
Mean proportion (%)
Difference in mean proportions (%)
p-value (corrected)
Rs
Sc
Function
95% confidence intervals
Amidase clustered with urea ABC transporter and ni...
Periplasmic nitrate reductase component NapE
Nitrogenase (molybdenum-iron)-specific transcripti...
Respiratory nitrate reductase beta chain (EC 1.7.9...
Nitric-oxide reductase (EC 1.7.99.7), quinol-depen...
Nitrite reductase [NAD(P)H] large subunit (EC 1.7....
Respiratory nitrate reductase alpha chain (EC 1.7....
8.00e-3
0.023
0.030
0.032
0.035
0.037
0.039
0.0
4.1
−2.5 −2.0 −1.5 −1.0 −0.5 0.0
Mean proportion (%)
Difference in mean proportions (%)
p-value (corrected)
Phosphorus
Rs
Sc
Level 3
95% confidence intervals
Phosphate_metabolism
4.73e-3
0.0
61.4
0 1 2 3 4 5 6 7
Mean proportion (%)
Difference in mean proportions (%)
p-value (correct
Rs
Sc
Function
95% confidence intervals
Pyrophosphate-energized proton pump (EC 3.6.1.1)
Soluble pyridine nucleotide transhydrogenase (EC 1...
Polyphosphate kinase (EC 2.7.4.1)
"Metal-dependent hydrolases of the beta-lactamase ...
Alkylphosphonate utilization operon protein PhnA
Phosphate transport system permease protein PstA (...
1.06e-3
9.08e-3
0.012
0.026
0.033
0.036
0.0
30.5
−4 −2 0 2 4 6 8
Mean proportion (%)
Difference in mean proportions (%)
p-value (corrected)
Potassium
Rs
Sc
Level 3
95% confidence intervals
Potassium_homeostasis
Glutathione-regulated_potassium-efflux_system_and_...
8.10e-3
0.020
0.0
97.6
−2.5 −2.0 −1.5 −1.0 −0.5 0.0 0.5 1.0 1.5 2.0
Mean proportion (%)
Difference in mean proportions (%)
p-value (correcte
Rs
Sc
Function
95% confidence intervals
Trk system potassium uptake protein TrkG
FKBP-type peptidyl-prolyl cis-trans isomerase FkpA...
Potassium voltage-gated channel subfamily KQT
Trk system potassium uptake protein TrkA
Potassium-transporting ATPase B chain (EC 3.6.3.12...
6.86e-3
0.016
0.024
0.029
0.045
0.0
22.2
−15 −10 −5 0 5 10
Mean proportion (%)
Difference in mean proportions (%)
p-value (corrected)
Sulfur
Rs
Sc
Level 3
95% confidence intervals
Release_of_Dimethyl_Sulfide_(DMS)_from_Dimethylsul...
0.025
0.0
0.1
−0.06 −0.05 −0.04 −0.03 −0.02 −0.01 0.00 0.01
Mean proportion (%)
Difference in mean proportions (%)
p-value (correct
Rs
Sc
Function
95% confidence intervals
Adenylylsulfate kinase (EC 2.7.1.25)
Taurine-binding periplasmic protein TauA
Sulfate adenylyltransferase, dissimilatory-type (E...
Dimethylsulfoniopropionate (DMSP) acyl CoA transfe...
Phosphoadenylyl-sulfate reductase [thioredoxin] (E...
1.80e-3
0.013
0.023
0.025
0.028
0.0
6.0
−0.2 0.0 0.2 0.4 0.6 0.8 1.0 1.2 1.4 1.6
Mean proportion (%)
Difference in mean proportions (%)
p-value (corrected)

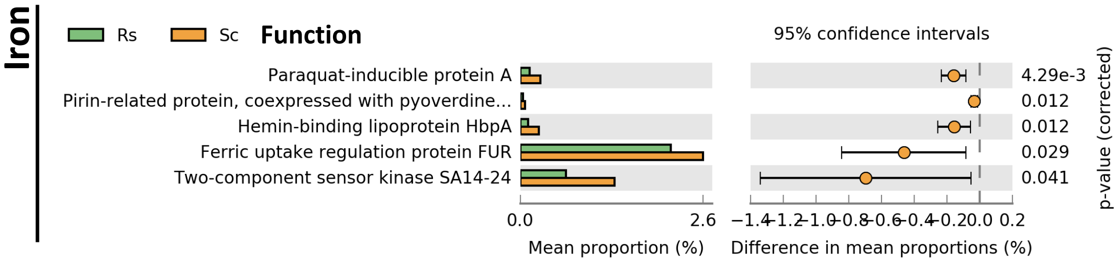

Supplementary Figure 3. Differential expression of metatranscriptome sequences affiliated to the metabolism of nutrients using the SEED database at level 3 and function. The differences between the treatments are based on Welch's t-test with Benjamini-Hochberg correction (*P* < 0.05). Bulk = bulk soil; Rs = Resistant cultivar; Sc = Susceptible cultivar.
